# A miniaturized microstrip antenna with tunable double band-notched characteristics for UWB applications

**DOI:** 10.1038/s41598-022-24384-2

**Published:** 2022-11-16

**Authors:** Zhang Chao, Zhao Zitong, Xiao Pei, Yu Jie, Liu Zhu, Li Gaosheng

**Affiliations:** 1 College of Electrical and Information Engineering, Hunan University, Changsha, 410082 China; 2 School of Physics and Electronics, Hunan Normal University, Changsha, 410081 China; 3 Kenbotong Technology Co., Ltd, Foshan, China; 4 School of Physics and Information, Fuzhou University, Fuzhou, 350108 China

**Keywords:** Electrical and electronic engineering, Applied physics

## Abstract

This paper proposes the step-by-step design procedure for obtaining independent dual band-notch performance, which provides a valuable method for designing tunable dual band-notched UWB antenna. The proposed antenna consists of the semicircle ring-like radiating patch with an elliptical-shaped slot and double split ring resonators on the top surface of the substrate and defected ground structure (DGS) on the bottom surface of the substrate. The operating frequencies ranged from 1.3 to 11.6 GHz (S11 < − 10 dB). By loading varactor diodes at the gap of the resonators structure and changing the varactor diode’s reverse bias voltage(0–30 V), a wider band-notched tuning range from 2.47–4.19 to 4.32–5.96 GHz can be achieved, which covers the whole WiMAX band and WLAN band. The experimental results agree well with the simulated results. The notched gain at notched frequency points is about − 5.3 dBi and − 5 dBi, demonstrating that the narrow-band interference signal could be efficiently suppressed. The security of UWB communication systems can be further enhanced. Meanwhile, the selection of varactor diode and DC bias circuit are fully considered. Hence, the accuracy of the experiment results and antenna operating performance have been improved. Furthermore, the proposed antenna only has an electrical size of 0.26λ*0.19λ at 1.3 GHz. Compared to the related reported antennas, the proposed antenna has achieved a simpler structure, low profile, compact size, tunable dual band-notched characteristics, extensive independent tunable range, and good band-notched performance simultaneously, to the best of our knowledge. The proposed antenna is believed to have a valuable prospect in UWB communication, Wireless Body Area Network, Industry Science Medicine, mobile communication applications, etc.

## Introduction

With the development of wireless communication technology, the service quality of wireless communication should be improved to meet increasing complicated functional communication demands. UWB communication system has gradually attracted extensive attention due to the numerous advantages of high bandwidth, high date rate, large capacity and multi-service^1^. UWB systems have many potential applications including IoT^2^, radar systems^3^, WBAN^4^ and medical application^5^. In 2002, the Federal Communications Commission(FCC) of the United States have allocated the 7.5 GHz UWB frequency spectrum which range from 3.1 to 10.6 GHz^6^. Antenna is an important component in the wireless communication system. High performance UWB antenna plays a very important role in UWB communication system^7^. The UWB antennas are mainly divided into dielectric resonator antennas (DRA)^8,9^, slot antenna^10,11^, Vivaldi antenna^12,13^, monopole antenna^14,15^ and other microstrip patch antenna^16–18^, etc. Among these design methods, monopole microstrip antennas have gradually obtained the widespread attentions due to unique characteristics of compact size, light weight, low profile, low cost, easy fabrication and conformal nature^19^.

However, although UWB antennas have a highly reliable quality, several narrow frequency bands can interfere with the UWB performance since it can inevitably affect the transmission spectrum, reliability and quality, such as WiMAX band (3.3–3.7 GHz), WLAN frequency band (5.15–5.825 GHz). In order to avoid the frequency coexistence interference from these undesired narrow band signals, the antenna with band-notched characteristics has attracted much attention in both academic and industrial communities of telecommunications since UWB frequency spectrum from 3.1 to 10.6 GHz has been allocated by U.S. Federal Communications Commission (FCC). The UWB antennas with band-notched characteristics are necessary. In recent years, a lot of UWB antennas with band-notched characteristics have been investigated in detail using various design methods^19–34^. The notched characteristics with UWB antennas can be achieved by etching various shaped slot on the radiating patch or ground plane^19–23,27,34^, incorporation of electric resonator resonator(ERR)^24^, split ring resonators(SRRs)^23,25,30,33^ or complimentary-split-ring-resonators(CSRRs)^26^, using parasitic elements^27–30^ and EBG resonators^20,31,32^, etc. However, it is not always convenient or efficient to control the notched frequency bands of UWB antenna by changing its shape or adding addition resonator structures.

Nowadays, the related research agendas towards shifting to realizing switchable, tunable and nonlinear features to avoid fabricated errors and to satisfy complex multifunctional requirements in real-time control systems^35–37^. Dynamically controlled band-notched UWB antenna have been considered as a suitable method to mitigate several important weaknesses of traditional band-notched UWB antennas (i.e. low flexibility, narrow notched-bands, weak adaptability) in complex wireless communication environment. Hence, it is urgent to design UWB antenna with dynamically switchable or tunable band-notched characteristics to solve those problems in practical application. The multiband antenna with reconfigurable and tunable characteristics have been implemented through using PIN diodes^35–38^, varactor diodes^39–43^ and other active components(i.e. capacitors^44,45^, FETs^46^ and MEMS switches^47^). In^35^, a flexible and frequency-reconfigurable antenna with harmonic suppression have been proposed for multiple wireless applications, which produced various different frequency bands by utilizing PIN diodes, Meanwhile, the unwanted high-order mode interference signal has been effectively suppressed by introducing filter stub to the feedline. In^36^, a miniaturizated multiband antenna with conformal and flexible characteristic has been demonstrated for flexible and smart portable devices. By introducing a stub in the circular patch and manipulating the switching state of the PIN diode, the antenna can achieve the operation frequency reconfigurability In^37^, a compact monopole antenna with flexible and frequency and pattern reconfigurable characteristics has been proposed for heterogeneous applications (such as GSM, ISM, 3G, 4G and LTE). The reconfigurable characteristics have been realized by the presence or absence of L-shaped stubs, which have been achieved by controlling the “ON” or “OFF” state of the PIN diode. In^38^, a flexible frequency reconfigurable multiband antenna has been exhibited by the activation and manipulation of the different modes. The dual-band mode and tri-band mode can be achieved by utilizing pin diode across the rectangle and semi-circular slot.

These flexible reconfigurable antennas have made excellent contributions to the further development of miniaturized reconfigurable antennas by introducing suitable slots and controlling the bias voltage on PIN diodes, while maintaining the miniaturization characteristics of the antenna, the reconfigurability of the frequency and pattern have been achieved, which has greatly expanded its range of applications. However, these reconfigurable antennas only focus on their operating band transition from one state to another, and do not focus on the antenna's ability to suppress interfering signals in the unwanted frequency band.

Moreover, the unwanted frequency band cannot be continuously controlled to cope with the increasing complicated communication environment, this is because that the PIN diodes can only be switched between two different states, one is “OFF” state, while another is “ON” state. This inherently drawback has limited the application in practical wireless communication. In order to change the notched-band frequency continuously, several band-notched UWB antenna using varactor diodes have been investigated^39,40,42,43^. Compared to PIN diodes, the varactor diodes can be used to achieve independent electronic control of notched band frequency by varying the reverse bias DC voltages of varactor diodes. Furthermore, the combination of PIN diode and varactor diodes are also used in UWB band-notched antenna^42^. To the best of our knowledge, most of these related research works have achieved reconfigurable bands by using PIN diodes or varactor diodes, etc. However, in most cases, the band notches cannot be independently controlled and the tuning range is relatively narrow, which decrease interference suppression capability. In addition, the type of varactor diode and the DC bias circuit is not discussed in depth, which may lead to inaccurate measurements. Some notched antenna has achieved notched characteristic by using slot resonator, but the radiation efficiency of notch frequency is not considered.

To achieve the wider and multiple independent tunable frequency spectrum, the stronger interference suppression capability, more accurate measurement results and further reduce the electrical size, we have demonstrated a compact independent tunable dual band-notched UWB microstrip antenna by only loading varactor diodes. Our work not only achieves multiple unwanted frequency bands (narrowband interference) in UWB communication system, but also these frequency bands can be dynamically tuned in real time by using varactor diodes, which allow better suppression of narrowband signals (ie. WLAN and WiMAX band) by introducing appropriate varactor diodes in UWB communication system. The step by step design method for obtaining independent dual band-notch performance and compact characteristics provides a valuable way of thinking for rapid tunable UWB band-notched antenna designs. The wider notched frequency band (2.47–4.19 GHz, 4.32–5.96 GHz) can be controlled continuously by adjusting the reverse bias voltage across the varactor diode, which can cover the whole WiMAX band and WLAN band. Both simulated results and experiment results verify that these interference narrowed bands can be effectively suppressed. The realized gain at the notched frequencies of the proposed antenna is about − 5.3dBi and − 5.09dBi, which demonstrated that the radiation efficiency of notch frequency can be suppressed effectively and enhance the security of UWB communication systems. In addition, the selection of varactor diode and the DC bias circuit are further discussed, which will enhance the operating performance and the accuracy of measurement results. The proposed tunable dual band-notched UWB microstrip antenna is supposed to be a good candidate for UWB wireless communication, ISM and WBAN, etc.

## UWB microstrip antenna design and results

**Figure 1:**
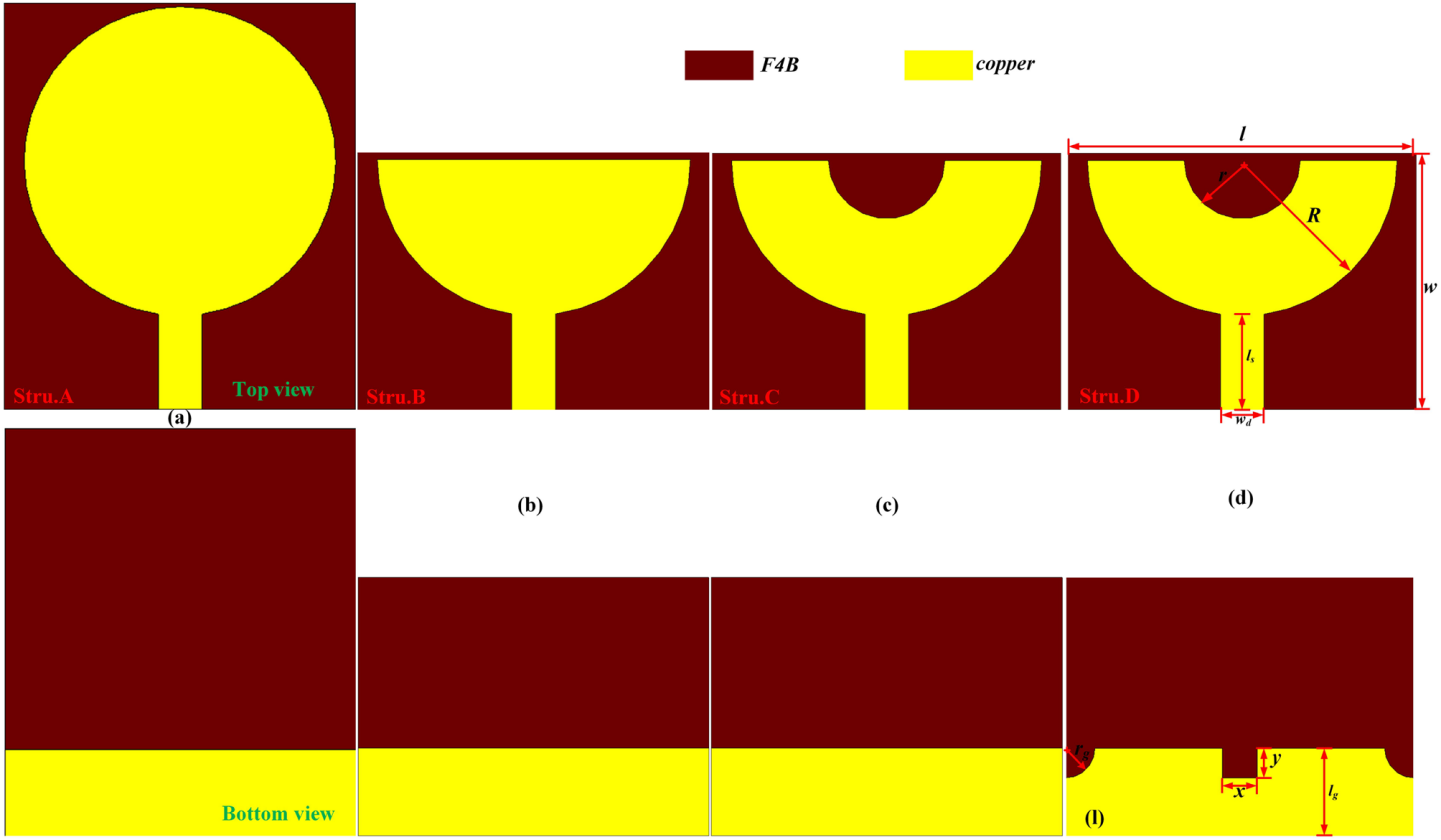
(**a**) The schematic diagram of the proposed UWB monopole microstrip antenna where there named as (**a**) Stru.A, (**b**) Stru.B, (**c**) Stru.C and (**d**) Stru.D.

The proposed compact UWB microstrip antenna is shown in Fig. 1, which is fabricated on the F4B dielectric substrate with thickness of 3.048 mm and relative permittivity of 2.94. Its loss tangent is 0.001. The electrical dimension of the proposed antenna is 0.26λ*0.19λ at 1.3 GHz. The antenna is composed of a semicircle ring-like radiating patch on the top layer of the substrate and a DGS with truncated edge corner structures and a rectangle slot on the bottom layer of the substrate, which have been excited by a 50 Ω microstrip feeding line. The width of microstrip feedline can be calculated by the Eqs. (1) and (2).

1 $$ Z_{0} = \frac{{Z_{0} }}{{\sqrt {\varepsilon_{eff} } \left[ {1.393 + \frac{w}{h} + \frac{2}{3}\ln \left( {\frac{w}{h} + 1.444} \right)} \right]}} $$

2 $$ \varepsilon_{{{\text{eff}}}} = \frac{{\varepsilon_{r} + 1}}{2} + \frac{{\varepsilon_{r} - 1}}{2}\left(1 + 12\frac{h}{w}\right)^{{ - \frac{1}{2}}} $$

where *Z*_*0*_ is the free space wave impedance. *Ɛ*_eff_ is the effective dielectric constant of the dielectric substrate. h is thickness of dielectric substrate. *w* is width of microstrip feedline. *Ɛ*_*r*_ is the relative permittivity of the dielectric substrate. The value of width of the microstrip feedline is around 7.5 mm. The other main designed parameters of the proposed antenna structure are given in Table 1.

**Table 1:** Main parameter of the proposed notch band antenna.

Parameter	Value(mm)	Parameter	Value(mm)
*R*	26.4	*l*	44.1
*r*	10	*w*	60
*l*_s_	16.4	*w*_d_	7.47
*l*_*g*_	15	*r*_g_	5
*x*	6	*y*	5

**Figure 2:**
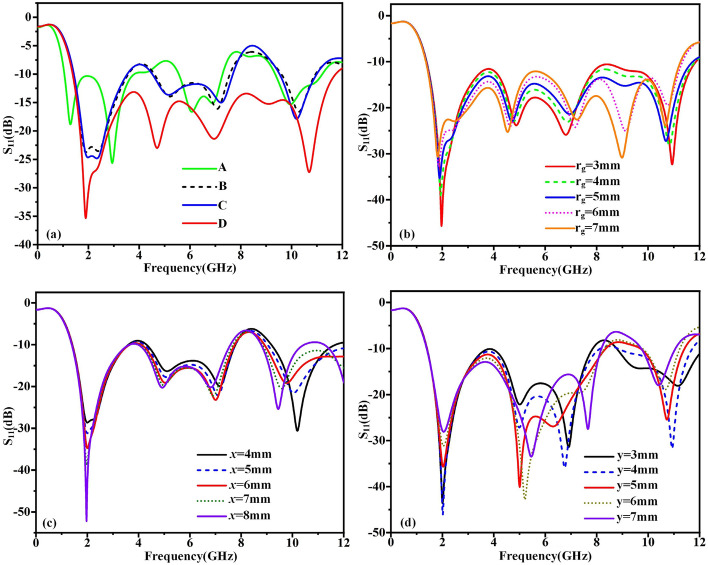
(**a**)The reflection coefficients of Stru.A, Stru.B, Stru.C and Stru.D, respectively. (**b**–**d**) The UWB performance optimization of Stru.D.

The step by step design procedure of UWB microstrip antenna based on traditional monopole antenna has been depicted in Fig. 1a–d. The simulated reflection coefficients of these antennas are acquired with the help of commercial software CST 2020, as is showed in Fig. 2. First of all, the classic microstrip fed printed circular disc UWB monopole microstrip antenna have been exhibited in Fig. 1a. This antenna structure is denoted as structure A for convenience. It can be seen from the Fig. 2a that the structure A can produce wide bandwidth as expected. Secondly, A new monopole microstrip antenna is designed by truncating semicircular-shaped patch based on structure A, as shown in Fig. 1b, denoted as structure B. From the Fig. 2a, it can be seen that structure B also have similar wide bandwidth performance compared with structure A. Thirdly, in order to reduce the manufacturing cost, a small semicircular-shaped patch have been further truncated based on structure B, as shown in Fig. 1c, denoted as structure C. As we can see from Fig. 2a, the good impedance matching performance of structure C still maintain without influences compared with structure B. Furthermore, in order to improved impedance matching performance and expand the bandwidth. By introducing the edge corner truncation and rectangle slots at the DGS based on structure C, the final UWB monopole microstrip antenna has been achieved, which is shown in Fig. 1d, denoted as structure D for convenience. The UWB performance optimization have been carry out by changing geometric structure parameters of DGS, as is shown in Fig. 2b–d. It can be seen that the wide-band characteristics of structure D have been further improved. The structure D has a very wide operating bandwidth range from 1.37 to 11.8 GHz(S_11_ < − 10 dB). The electrical size of final UWB antenna structure D is only 0.26λ*0.19λ at the 1.37 GHz.

## Tunable dual band-notched UWB microstrip antenna design, results and discussion

**Figure 3:**
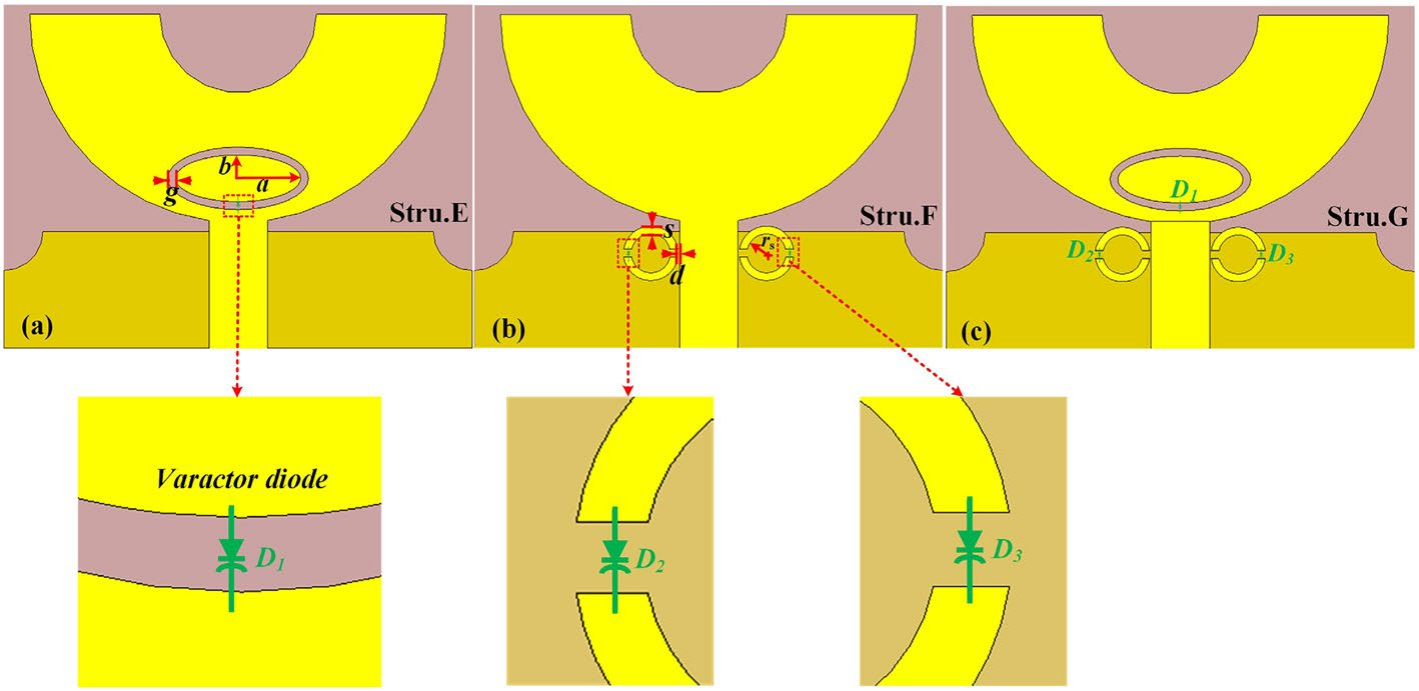
(**a**) The schematic diagram of the proposed UWB loaded with varactor diode with tunable dual band-notched characteristics where there named as (**a**) Stru.E, (**b**) Stru.F and (**c**) Stru.G.

In order to achieved electronically controllable dual band-notched characteristics of the proposed UWB microstrip antenna. A novel UWB microstrip antenna with tunable band-notched characteristics has been proposed based on structure D which is shown in Fig. 3c, denoted as structure G for convenience. As you can see from Fig. 3c, The propose dual band-notched UWB antenna is composed of a semicircle ring-like radiating patch with an elliptical-shaped slot and dual SRRs near to microstrip feedline on the top layer of the substrate and a defected ground structure(DGS) with truncated edge corner structures and a rectangle slot on the bottom layer of the substrate. Further, by embedding three varactor diodes to the elliptical-shaped slot and dual SRRs on the structure G, the independent tunable dual band-notched characteristics have been achieved. The optimal parameters of an elliptical-shaped slot and SRRs are *a* = 8 mm, *b* = 3 mm, *g* = 1 mm, *r*_*s*_ = 2.5 mm, *d* = 0.2 mm, *s* = 1 mm, respectively. The step-by-step design procedure of the proposed UWB microstrip antenna with tunable band-notched characteristics have been depicted in Fig. 3a–c. It provides a valuable way of thinking for rapid tunable UWB band-notched antenna designs. The simulated VSWR performance of these antennas has been shown in Fig. 5.

**Figure 4:**
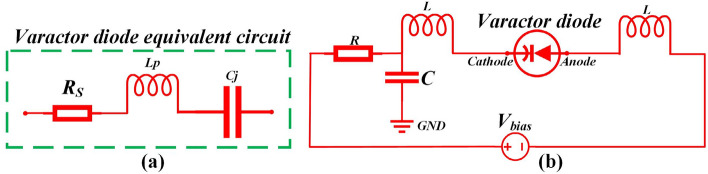
(**a**) The simple equivalent circuit of varactor diode (Skyworks SMV 1405-079LF), (**b**) The DC bias circuit for varactor diode.

**Figure 5:**
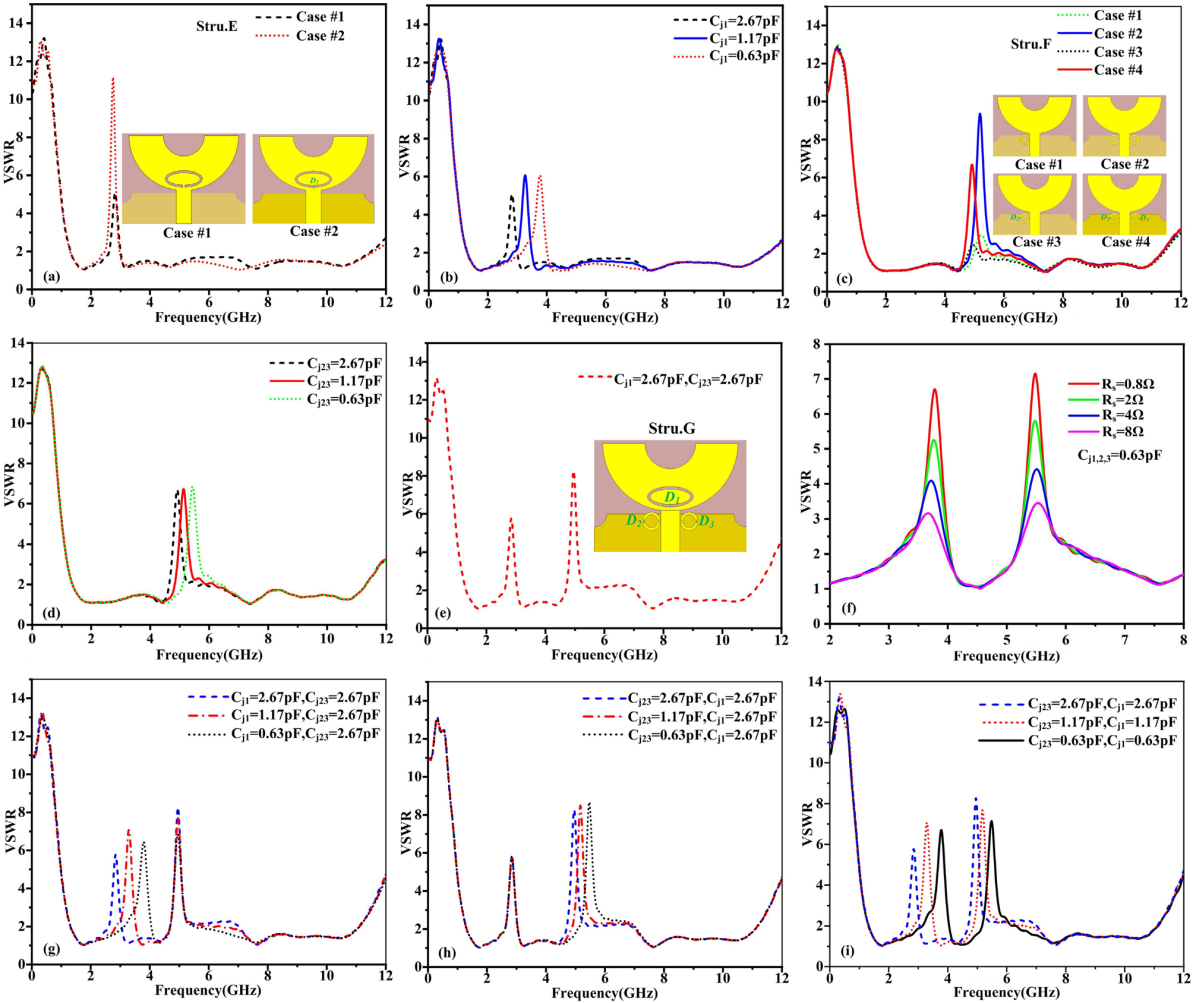
The VSWR characteristics of structure E, (**a**) for two different cases, (**b**) performance at C_j1_ variation. (**c**) The VSWR characteristics of structure F for four different cases, (**d**) performance at C_j23_ variation. The VSWR characteristic of structure G, (**e**) when junction capacitance of D_1_–D_3_ at 2.67 pF, (**f**) when the series resistance R_s_ vary, (**g**) performances at C_j1_ variation, (**h**) performances at C_j23_ variation and (**i**) performances at C_j1_ and C_j23_variations.

First of all, A single band-notched UWB microstrip antenna have been designed by introducing elliptical-shaped slot in the semicircular-shaped radiation patch and placing a varactor diode (Skyworks SMV 1405-079LF) in the gap of the slot. This antenna structure is denoted as structure E for convenience. The equivalent circuit model and circuit parameters of the varactor diode are further studied. The simple equivalent circuit of the varactor diode is the RLC series model in CST and it is displayed in Fig. 4a, *R*_*s*_ is the series resistance, *C*_j_ is the junction capacitance in which we more interested. *L*_*p*_ is package inductance. According to the datasheet of Skyworks SMV 1405-079LF, the typical value of *R*_*S*_ and *L*_*p*_ are 0.8Ω and 0.7nH for the varactor diode. The junction capacitance *C*_j_ change in the simulations and the values are selected as 2.67pF, 1.17pF and 0.63pF corresponding to the reverse bias voltage of 0 V, − 5 V and − 30 V, respectively. In addition, the selection of suitable varactor diode have an important influence on the performance of proposed antenna. The main reason is that the parasitic parameters (particularly the series resistance R_s_) affect the impendence matching and the detailed analysis have been discussed later(see Fig. 5f). What's more, in order to maintain a more stable working state, The DC bias network which is necessary for varactor diodes, as is shown in Fig. 4b. As you can see, the value of resistance is set 200Ω, which is used for suppress the excess current of varactor diode. The large DC blocking capacitor is set 1000 pF here so that it could eliminate high frequency harmonics of DC power supply. In addition, the two inductors (L = 0.1 uH) are used as RF chokes to prevent the RF signal passing through the DC bias circuit.

The structure E have been designed in two cases (case#1, case#2) from inserting the metallic strip and a varactor diode into the slit, respectively. The VSWR characteristics of two cases of the structure E are illustrated in Fig. 5a, It can be seen that the similar band-notched characteristics of antenna have been achieved, covering frequency range between 2.48 and 2.97 GHz. It is worth to note that there are a few resonant frequencies and magnitudes deviations of VSWR between the case #1 and case #2, since the effects of parasitic parameter and additional ohmic losses of the varactor diode cannot be neglected. For case #1, the notch frequency of elliptical-shaped slot can be calculated using

3 $$ f_{{{\text{notch}}}} = \frac{c}{{2L\sqrt {\varepsilon_{eff} } }} $$

where *L* is the whole length of the elliptical-shaped slot, *Ɛ*_*eff*_ is the effective relative permittivity. c is the speed of light in free space. The VSWR performances of the structure E are plotted in Fig. 5b when the junction capacitance of varactor diode varies. Here, the tunability of 2.61 GHz to 4.01 GHz has been achieved by changing junction capacitance of varactor diode from 2.67 to 0.63pF. That is to say, by controlling the junction capacitance of varactor diode, the independent tunable band-notched characteristics can be achieved. Then, another single band-notched UWB microstrip antenna have been demonstrated by introducing double SRRs near the edge of microstrip feedline and loading varactor diodes across the gaps cut in the SRRs. The antenna structure is denoted as structure F for convenience. Similarly, the band notched characteristics of structure F have been investigated in four cases (case#1, case#2, case#3, case#4). As shown in inset in Fig. 5c. Here firstly a complete C-shaped SRR has been excited by the microstrip feedline (case #1). For case #1, the notch frequency of C-shaped SRR can be evaluated as

4 $$ f_{{\text{n}}} = \frac{1}{{2\pi \sqrt {LC_{{{\text{eq}}}} } }} = \frac{1}{{2\pi \sqrt {L_{T} \left[ {\frac{{(\pi r_{0} - g)\sqrt {\varepsilon_{e} } }}{{2c_{0} Z_{0} }} + \frac{{\varepsilon_{0} ch}}{{2{\text{s}}}}} \right]} }} $$

5 $$ L_{{\text{T}}} = 0.00508l(2.303\log_{10} \frac{4l}{d} - 2.451) $$

where *C*_*eq*_ is equivalent capacitance of the C-shaped SRR structure and *L*_*T*_ is total inductance of the SRR, *c* is light speed of free space. *Ɛ*_*e*_ is the effective permittivity of dielectric and Z_0_ is the impendence of dielectric. *r*_*0*_ is the radius of the circle SRR and s is the width of the gap of the SRR. Secondly, double C-shaped SRRs have been introducing near the edge of feedline(case #2),Next, the partial metallic strip of single SRR is removed and loaded with the varactor diode(case #3).

Finally, the double SRRs with defected metal are loaded with varactor diodes(case #4). The VSWR characteristics of four cases of the structure F are shown in Fig. 5c. As you can see, here in case #1, the notch frequency is producing at 5.23 GHz. In case #2, the band-notched characteristics have been improved with similar notch frequency and the VSWR value of notch frequency is increased compared with case #1. In case #3 and case #4, it can be seen that the VSWR performance is similar to the case #1 and case #2 with a little frequency deviations and lower magnitude, since the varactor diode cannot be completely equivalent to short circuit. Furthermore, By changing the junction capacitance of varactor diode across the gap of the double SRRs, Another single tunable notched band covers WLAN band has been achieved, as shown in Fig. 5d. We can observe that the notch frequency change from 4.92 to 5.12 GHz and 5.44 GHz when C_j1_ changes from 2.67pF to 1.17pF and 0.63pF in the simulation. It is demonstrated that the independent tunable notch frequency band can be realized by varying the junction capacitance of varactor diode. Finally, now, structure E an structure F are merged to form another antenna which is consider as structure G for suppressing two bands simultaneously, as shown in Fig. 5e. The influences of the series resistance R_s_ on band-notched performance have been studied. It has been found that the magnitudes of VSWR are mainly affected by the series resistance R_s_, which are shown in Fig. 5f. It can be seen that the magnitudes of VSWR decrease when the series resistance R_s_ is increased, indicating that the ohmic losses affect the magnitudes of VSWR. That is to say, choosing a varactor diode with a smaller series resistance will help improve the notch performance, which provides a valuable method for selecting appropriate varactor diode for a high performance tunable notch antenna. The tunable band-notched characteristics of structure G also have been studied by controlling different varactor diodes. As shown in Fig. 5g, it seems here that by tuning the junction capacitance of varactor diode D_1_ only, the notch frequency is changing from 2.84 to 3.28 GHz and 3.79 GHz while maintaining a second notch at a fixed frequency at 4.95 GHz. Again, the notch frequency is varying from 4.95 to 5.16 GHz and 5.47 GHz while maintaining the first notch fixed frequency at 2.84 GHz by varying the junction capacitance of varactor diode D_2_ and D_3_ and it is plotted in Fig. 5h. Further, as shown in Fig. 5i, the tunable dual band-notched characteristics have been achieved from 2.84 to 3.29 GHz and 3.78 GHz and 4.95 GHz to 5.17 GHz and 5.48 GHz at simultaneous combinations of the junction capacitances of D_1_–D_3_ between 2.67 and 0.63pF, which imply that the independent tunable dual band-notched characteristics can be achieved simultaneously and independently.

**Figure 6:**
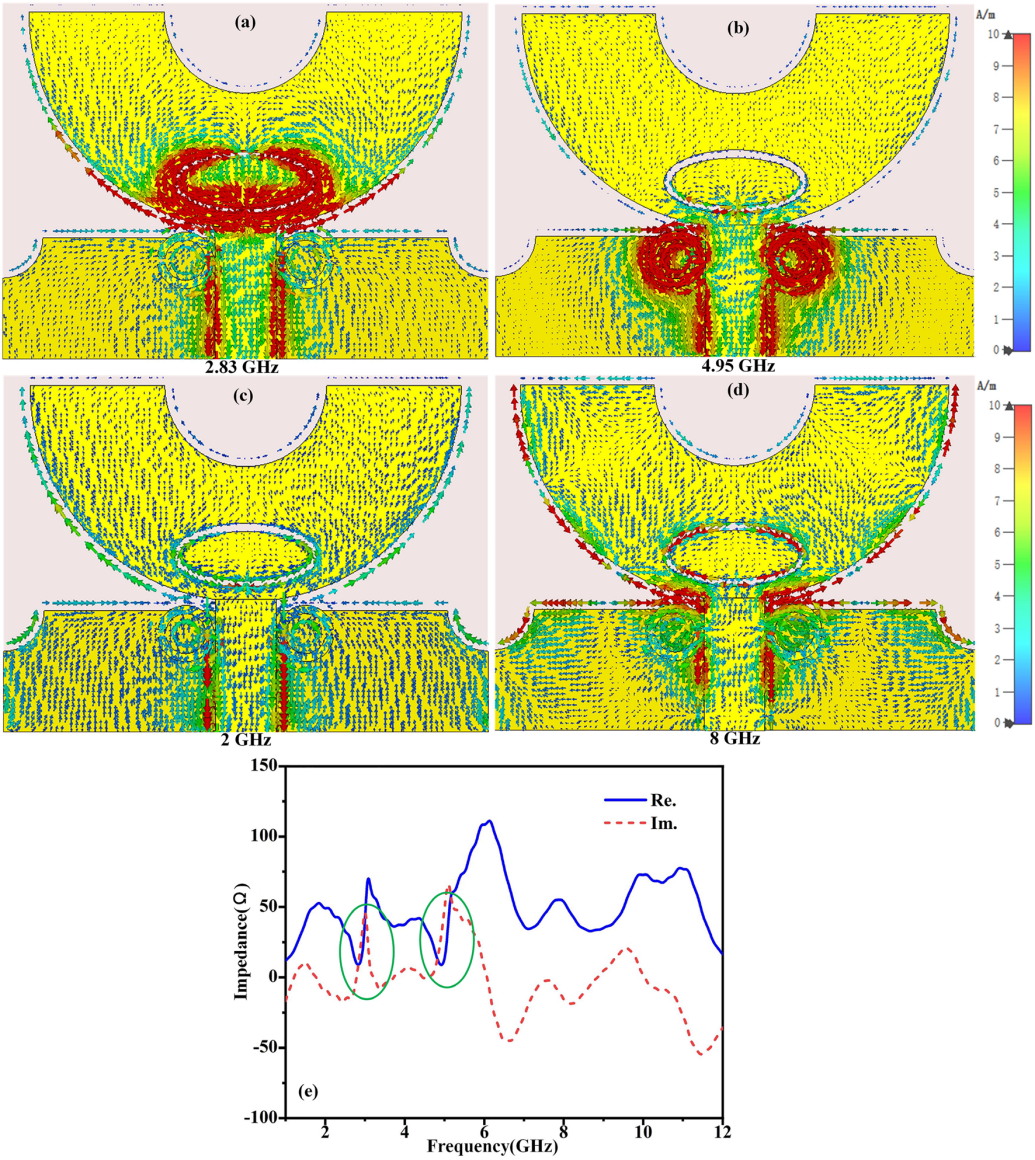
(**a**–**d**) Distribution of surface current of the structure G at 2.83 GHz, 4.95 GHz, 2 GHz, and 8 GHz, respectively. (**e**) The impedance of the antenna.

To better understand the performance of the proposed antenna with dual band-notched characteristics, the simulated surface current distributions of proposed antenna at notched frequencies (i.e. 2.83 GHz, 4.95 GHz) and pass-band frequencies (i.e. 2 GHz, 8 GHz) have been observed in Fig. 6a–d. It can be seen from Fig. 6a that the surface current concentrate on the elliptical-shaped slot in the semicircle ring-like radiating patch at 2.83 GHz and the electromagnetic energy cannot be radiated into free space, therefore, the first notch frequency has been produced. Similarly, the simulated surface distributions at the second notch frequency (4.95 GHz) have been illustrated in Fig. 6b. It can be seen that the strong surface current concentrate on double SRRs near the microstrip feedline. What’s more, the impedance of central frequency of the second notch frequency band is close to zero when the length of the surface current path on SRR is equal to 1/4 wavelength at center frequency of notch-band. Hence the second notch band has also been aroused. Especially, it is worth to note that the notched characteristic have been enhanced by high concentration of the surface current around the slot resonator structure and double SRR structure. The simulated surface current distributions at frequency points of pass-band (i.e.2 GHz, 8 GHz) have also been observed in Fig. 6c, d. It can be seen that the surface current is uniformly distributed to different areas of the radiating patch of antenna, which imply that this proposed antenna has a good radiating performance characteristic at these frequency points. Figure 6e depicts the simulated port impedance curve of the proposed antenna. It can be seen that real part of impedance and imaginary part of impedance is closed to zero in the notched bands centered around 2.83 GHz and 4.95 GHz, which can also effectively verify that the high impedance mismatches at notch frequency points and the electromagnetic energy can be effectively rejected.

## Experiment validation and discussion

**Figure 7:**
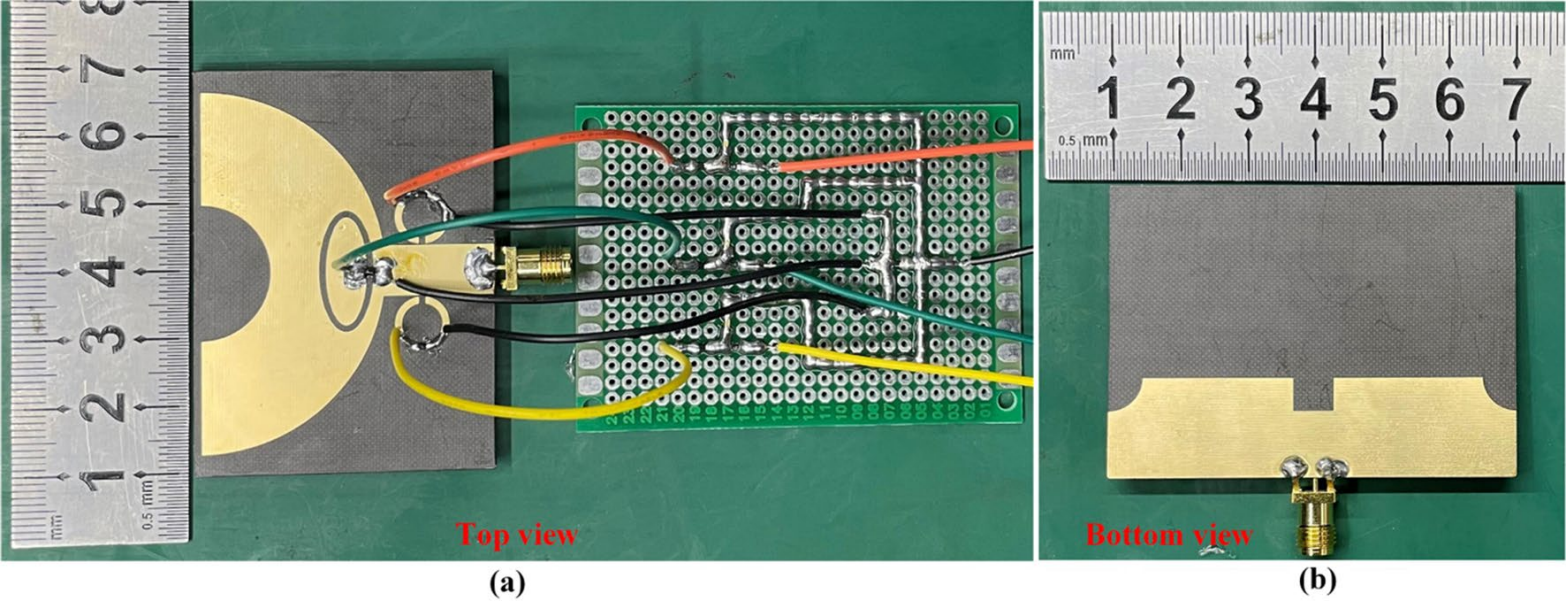
Fabricated prototype of antenna with its (**a**) top and (**b**) bottom view.

**Figure 8:**
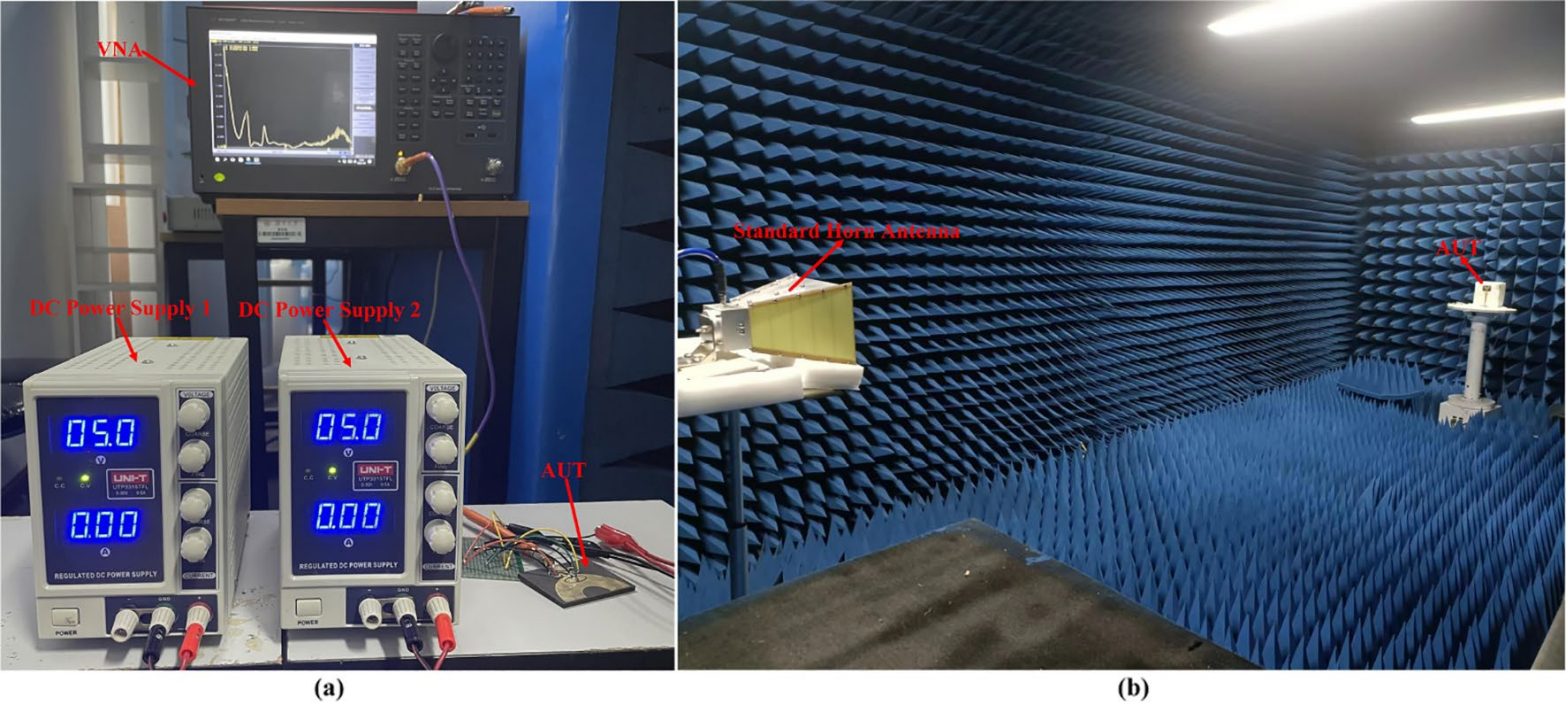
(**a**) Measurement of reflection coefficient of the proposed antenna with VNA. (**b**)Anechoic chamber.

In order to experimentally verify the dual tunable band-notched characteristics of proposed antenna loaded with varactor diodes, the top view and bottom view of fabricated sample are shown in Fig. 7. We can see that the black wire is connected to the negative terminal of DC power supply whereas three other color wires are connected to the positive terminal of DC power supply. The DC bias circuit has been installed on printed circuit board (PCB), which is necessary for measurement of antenna performance. The DC bias circuit could be used to eliminate high frequency harmonics of DC power supply and prevent the RF signal from passing through the DC bias circuit. Hence, the operating performance and the accuracy of measurement results will be improved efficiently. Figure 8a exhibited a vector network analyzer (VNA Agilent 5027A) which is connected to fabricated antenna sample was used for measuring the reflection coefficients and the regulated DC power supply (UNI-T UTP3315TFL) is used to provide DC bias voltage as much as 30 V. The far-field radiation characteristics of the antenna under test (AUT) have been obtained by anechoic chamber which is shown in Fig. 8b.

**Figure 9:**
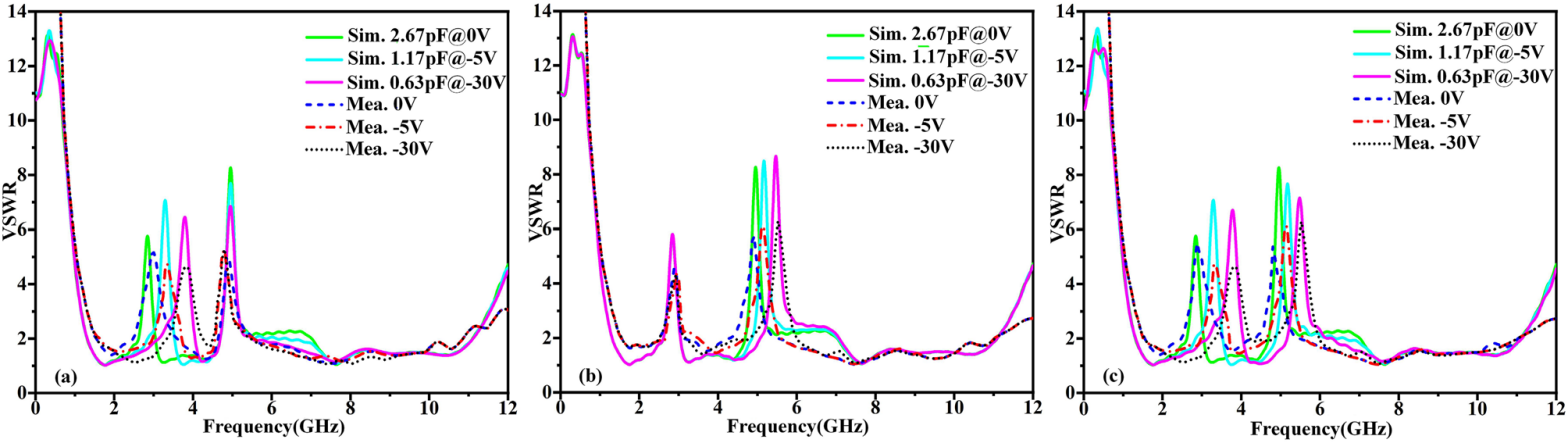
(**a**) Simulated and measured VSWR characteristics of structureG the voltage across the varactor diode (**a**) D_1_ varies(D_2_, D_3_@0 V), (**b**) D_1_ and D_2_ varies simultaneously(D_2_, D_3_@0 V), (**c**) D_1_, D_2_ and D_3_ varies simultaneously.

According to tunable band-notched characteristics of final structure G mentioned above, the measured VSWR characteristics for structure G have also been investigated by applying different bias voltages to varactor diodes. As is shown in Fig. 9a, the VSWR characteristics are measured by varying the different reverse bias voltage(i.e.0 V, − 5 V, − 30 V) applied to D_1_ while maintaining zero bias voltage for D_2_ and D_3._ According to datasheet of Skyworks SMV 1405-079LF, the junction capacitance 2.67pF, 1.17pF and 0.67pF correspond to 0 V, − 5 V, − 30 V. It can be seen that measured notch frequency changing from 3 to 3.84 GHz, while applying the voltage to D_1_ from 0 to − 30 V. As is shown in Fig. 9b, the VSWR characteristics are measured by varying the different reverse bias voltages (i.e. 0 V, − 5 V, − 30 V) applied to both D_2_ and D_3_ simultaneously while maintaining zero bias voltage for D_1_. It can be seen that the measured notch frequency changing from 4.92 to 5.52 GHz, while applying the voltage to D_2_ and D_3_ from 0 to − 30 V. As is shown in Fig. 9c, the VSWR characteristics are measured by varying the different reverse bias voltage (i.e. 0 V, − 5 V, − 30 V) applied to D_1_–D_3_ simultaneously, it can be seen that the measured notch frequency changing from 2.88 to 3.84 GHz and 4.8 GHz to 5.52 GHz, while applying the voltage to D_1_–D_3_ from 0 to − 30 V. In order to observe the tunable band-notched characteristics more clearly, the detailed comparison have been exhibited in Table 2. It is worth to be noted that the measured results agree well with simulated results with little frequencies deviations, since the diode parameter in simulation and measurement cannot be totally same and fabricated tolerances are inevitable.

**Table 2:** Measured and simulated notch frequency bands comparison.

D_1_(0 to − 30 V) D_2_&D_3_(0 V)		D_1_(0 V) D_2_&D_3_(0 to − 30 V)		D_1_(0 to − 30 V) D_2_&D_3_(0 to − 30 V)	
Sim. (GHz)	2.62–4.04	Sim. (GHz)	4.69–6.35	Sim. (GHz)	2.61–4.03; 4.67–6.28
Mea. (GHz)	2.47–4.24	Mea. (GHz)	4.32–5.94	Mea. (GHz)	2.47–4.19; 4.32–5.96

**Figure 10:**
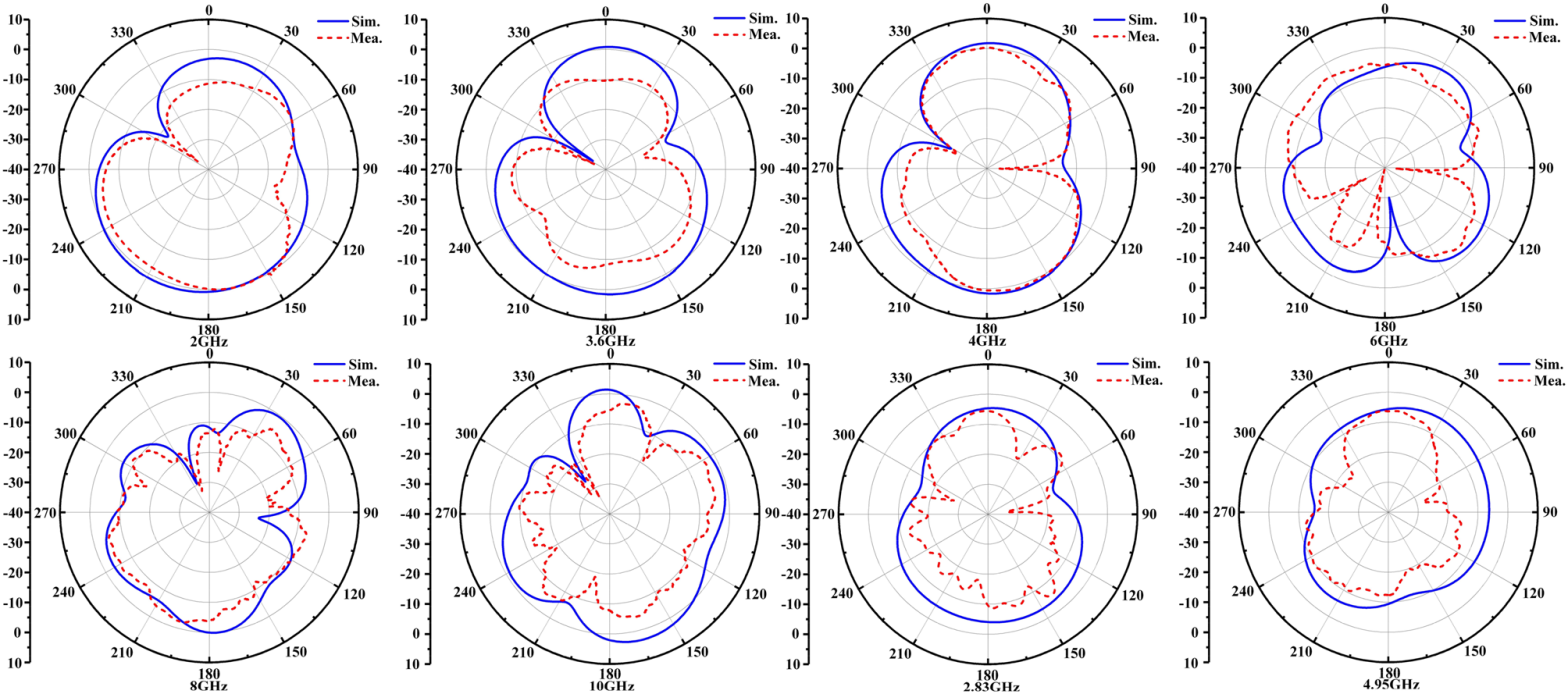
The simulated and measured E-plane radiation patterns.

In Fig. 10, we have plotted the simulated and measured E-plane far-field radiation pattern which located in 2 GHz, 3.6 GHz, 4 GHz, 6 GHz, 8 GHz, 10 GHz, 2.83 GHz and 4.95 GHz, respectively. It was been tested in the anechoic chamber. The measured realized gains are 1.34dBi, 1.7dBi, 1.85dBi, 3.1dBi, 4.52dBi and 5dBi at 2 GHz, 3.6 GHz, 4 GHz, 6 GHz, 8 GHz and 10 GHz, respectively. It can be seen that a near omnidirectional radiation patterns like a monopole antenna have been achieved, the measured results are agree well with simulated results with a little deviation, this may be due to fabricated tolerances, measured tolerances and the simulated and measured parameters difference of varactor diode, etc. It is worth to note that the radiation pattern does not remain omnidirectional at higher frequencies due to higher dielectric loss with increasing frequencies. In addition, the measured radiation pattern at notch frequencies (i.e.2.83 GHz, 4.95 GHz) suffer from some distortions, since nearly all the surface current is trapped at band-notch structures in the proposed antenna.

**Figure 11:**
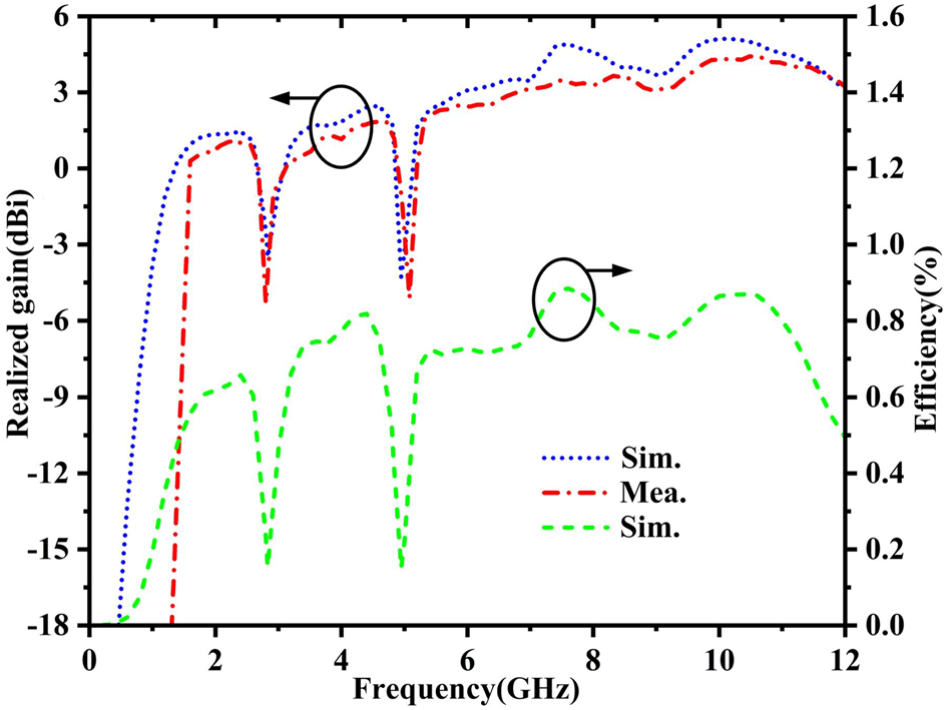
Realized gain comparison and simulated radiation efficiency of the proposed antenna.

Both simulated realized gain and the total radiation efficiency and measured realized gain of proposed antenna over the whole frequency range have been plotted in Fig. 11. It can be seen that the measured and simulated results are matched across the whole operating frequencies within an accept limit. For gain performance of antenna, the measured realized gain is changed from 0.95 to 4.42dBi over the full frequency range except for the notched band, which are lower than nearly 1dBi compared to simulated realized gain. It is mostly due to SMA connector loss, additional cable loss and ohmic loss of varactor diodes, etc.

The measured notch gains at notch frequencies are − 5.3dBi and − 5.09dBi, respectively, which are also lower compared to simulated notch gain. For simulated radiating efficiency of antenna, it can be seen that the measured radiation efficiency is over 60% and the max radiation efficiency can up to 88.4% across the whole operating frequencies except for the notch frequency band. Furthermore, the simulated total radiation efficiency at the notch frequencies are 15.4% and 15%, respectively. The simulated and measured results at notch frequency are in accordance with our expectations. The lower gain and lower efficiency at the notched frequency band of antenna has demonstrated that the narrowband interference (i.e. WiMAX band, WLAN band) has been effectively suppressed and enhanced the security of UWB communication system.

A performance comparison between previous reported related band-notched UWB antenna and this work have been exhibited in Table 3. Many reported related antennas(i.e. ^23,24^, etc.) have achieved band-notched characteristics by introducing multiple resonator structures and etching multiple slots. The band-notched characteristics of these reported works can only be tailored by tuning the geometrical parameters. However, it is not always convenient or practically useful to manipulate the notch frequency by changing its shape. Although the antenna in^48,49^ have a single tunable notch-band by using varactor diode, it cannot avoid the interference from other narrowband and the notch band tuning range is still narrow. The antennas in^25,50,51^ have realized dual band-notch characteristics by introducing PIN diode, but the band-notched antenna can only operate in two or three states, since the PIN diode only have two conditions (“ON” state and “OFF” state). The proposed antenna with switchable and tunable band-notched characteristics in^52^ has been achieved by mounting both Pin diodes and varactor diodes, but there exists only a single tunable notch band when pin diode switch condition at a time. The two notch-band cannot be achieved simultaneously. The antenna in^53^ has dual band-notched characteristics, but only a single notch band can be tuned. Closely related work related to this proposed antenna is exhibited in^43^, which have achieved dual tunable notched band by introducing varactor diodes, the dual notched band can be tunable simultaneously, but there still have some drawbacks, such as small band-notched tuning range, large electrical size, poor band-notched performance, etc.

**Table 3:** Comparation of the proposed tunable band-notched antenna and related designs.

Ref. (year)	Bandwidth (GHz)	Tuning range (GHz)	Switch type	Notch gain	Electrical size
2015^48^	3.08–10.5	5.07–5.83	Varactor	− 1.45	0.3λ*0.24λ
2019^49^	1.7–6	2–2.12	varactor	0.11	0.43λ*0.29λ
2017^50^	3.1–13	5.5, 7.4	PIN	− 4, − 6.1	0.31λ*0.25λ
2020^51^	3.18–12.2	3.5, 5.5	PIN	− 5.1, 5.2	0.4λ*0.28λ
2019^25^	2.2–14.6	3.5,5.5	PIN	− 2, − 2.5	0.3λ*0.23λ
2021^52^	2.8–9	4.2–4.8/5.8–6.5	PIN, Varactor	NR	0.34λ*0.24λ
2021^43^	2.6–14	5.5–5.9/7–7.4	Varactor	1.6	0.31λ*0.26λ
2022^53^	3.1–12.5	3.6/5.6–7.7	Varactor	− 4.1, − 4.9	0.38λ*0.27λ
This work	1.3–11.6	2.47–4.19 4.32–5.96	Varactor	− 5.3, − 5.0	0.26λ*0.19λ

λ is the guided wavelength at the lowest frequency of the operating band .

Hence, a tunable dual notched-band UWB microstrip antenna by using varactor diodes has been proposed in this paper. Compared to related antenna mentioned above, this proposed antenna has large tuning range, covering frequency range from 2.47 to 4.19 GHz(1.72 GHz) and 4.32 GHz to 5.96 GHz(1.64 GHz), respectively. Furthermore, the proposed antenna has UWB range of 1.3–11.6 GHz and the electrical size is only about 0.26λ*0.19λ at 1.3 GHz. The proposed antenna has advantages of more compact size, much wider tuning range, good band-notched characteristics and better gain characteristics either better or comparable with the other reported works in Table 3.

The novelty and contributions of the proposed notched antenna are that we have proposed a step-by-step design procedure for obtaining independent dual band-notch performance based on traditional monopole antenna, which provides a valuable way of thinking for rapid tunable UWB band-notched antenna designs. The equivalent impedance and notch frequency of band-stop resonator (elliptical-shaped slot resonator and C-shaped Split ring resonator) have been investigated by used resonance theory and empirical formula. Hence, the notched resonant frequency can be efficiently evaluated and calculated. Meanwhile, the notched characteristic has been enhanced by high concentration of the surface current around the slot resonator structure and double SRR structure, which enhanced the suppression capability of the narrowband interference signal. The varactor diode equivalent circuit model has been further analyzed. This is a fact that the selection of a varactor diode with a smaller series resistance will help improve the notch performance, which provides a valuable method to select appropriate varactor diode for high performance tunable notch antenna. The DC bias circuit has been used to eliminate high frequency harmonics of DC power supply and prevent the RF signal from passing through the DC bias circuit, improving the accuracy of measurement. Hence, the operating performance and the accuracy of measurement results will be improved efficiently. Moreover, the wider notched frequency band (2.47–4.19 GHz, 4.32–5.96 GHz) can be controlled continuously by adjusting the reverse bias voltage across the varactor diode, which can cover the whole WiMAX band and WLAN band. Compared with other related notch antennas, the proposed antenna has a simpler structure, lower profile, compacted size(0.26λ*0.19λ), easy integration, extensive independent tunable range, lower cost and better ability to suppress narrowband interference signals.

## Conclusions

The proposed antenna with tunable dual band-notched characteristics for UWB applications has been proposed in this paper. The band-notched performance and compacted characteristics have achieved by the step by step design procedure based on traditional monopole antennas. By placing varactor diode across the gap of C-shaped SRRs near to feedline and inserting the varactor diode into the gap of elliptical-shaped slot, the independent controllable and continuous tunable dual band notch characteristics are achieved for WiMAX band and WLAN band. The measured results agree well with simulated results. It is shown that a wider tunable dual notch-bands (2.47–4.19 GHz, 4.32–5.96 GHz) have been attained within the operating bandwidth of 10.3 GHz (1.3–11.6 GHz). The radiation efficiency and realized gain at notch frequencies is very low, which imply that the proposed antenna can efficiently suppress the narrowband interference originated from WiMAX frequency band and WLAN frequency band in the UWB communication systems. To improve the accuracy of measurement and enhance the antenna performance, the DC bias circuit has been used to eliminate high frequency harmonics of DC power supply and prevent the RF signal from passing through the DC bias circuit. In addition, a varactor diode with a smaller series resistance will help improve the notch performance, which provides a valuable method to select appropriate varactor diode for high performance tunable notch antenna. It is believed that the proposed antenna has potential in many application areas including the UWB wireless communication, ISM, WBAN, mobile communication applications, etc.

## Supplementary Information

Supplementary Information.

## Data Availability

The datasets generated during and/or analyzed during the current study are available from the corresponding authors (Zhu Liu and Pei Xiao) on reasonable request.
